# Transformer-Driven Affective State Recognition from Wearable Physiological Data in Everyday Contexts

**DOI:** 10.3390/s25030761

**Published:** 2025-01-27

**Authors:** Fang Li, Dan Zhang

**Affiliations:** Department of Psychological and Cognitive Sciences, Tsinghua University, Beijing 100084, China; li-f24@mails.tsinghua.edu.cn

**Keywords:** affective state recognition, transformer model, multi-modal data

## Abstract

The rapid advancement in wearable physiological measurement technology in recent years has brought affective computing closer to everyday life scenarios. Recognizing affective states in daily contexts holds significant potential for applications in human–computer interaction and psychiatry. Addressing the challenge of long-term, multi-modal physiological data in everyday settings, this study introduces a Transformer-based algorithm for affective state recognition, designed to fully exploit the temporal characteristics of signals and the interrelationships between different modalities. Utilizing the DAPPER dataset, which comprises continuous 5-day wrist-worn recordings of heart rate, skin conductance, and tri-axial acceleration from 88 subjects, our Transformer-based model achieved an average binary classification accuracy of 71.5% for self-reported positive or negative affective state sampled at random moments during daily data collection, and 60.29% and 61.55% for the five-class classification based on valence and arousal scores. The results of this study demonstrate the feasibility of applying affective state recognition based on wearable multi-modal physiological signals in everyday contexts.

## 1. Introduction

Wearable physiological measurement technology has advanced the field of affective computing by enabling natural and unobtrusive tracking of individuals’ affective states [1]. These developments offer new opportunities for applications in human–computer interaction, personalized mental health treatment, and adaptive learning systems. However, detecting affective states in everyday contexts remains challenging, due to the dynamic and transient nature of emotions, as well as the noise and variability inherent in long-term physiological recordings from real-world environments.

One major challenge lies in managing the continuous and dynamic physiological data collected during daily activities [2,3]. Signals such as heart rate, skin conductance, and electroencephalogram (EEG) exhibit complicated temporal patterns and interdependencies that are difficult to predict in uncontrolled, real-world settings. Another challenge is handling the complexities of multi-modal signals, as each modality provides distinct yet complementary information about affective states. Traditional machine learning methods, such as support vector machines [4] and random forests [5], have been employed for affective state recognition. However, these approaches often fail to capture intricate temporal dynamics and cross-signal relationships [6], primarily because they focus on features from independent signals, while neglecting their interdependencies. Advances in deep learning technology have partially addressed these limitations. Methods such as convolutional neural networks (CNN) and recurrent neural networks (RNN) have been developed to model sequential and spatial patterns in physiological data [7,8,9,10,11] and have been made to integrate complementary signals, such as autonomic activity from skin conductance and cardiac patterns, for affective state recognition [12,13,14]. Novel Transformer models [15] have successfully addressed similar challenges, such as long-term dependencies and cross-modal interactions, in fields like natural language processing and computer vision, yet their potential for real-world affective state recognition remains unexplored.

This paper presents a Transformer-based algorithm designed to address the challenges of real-world affective state recognition. Transformer models demonstrate remarkable efficacy in capturing long-term dependencies and cross-modal interactions, making them highly suitable for analyzing multi-modal physiological data in everyday settings. Our proposed framework leverages self-attention mechanisms to focus on the relevant features of each physiological signal, while capturing their complex interrelationships over time.

Utilizing the Daily Ambulatory Psychological and Physiological recording for Emotion Research (DAPPER) dataset [16], comprising five days of uninterrupted wrist-worn recordings of heart rate, skin conductance, and triaxial acceleration from 88 subjects, our Transformer-based methodology demonstrates the effectiveness of leveraging multi-modal wearable data for accurate affective state recognition in daily settings. The model’s performance in both binary and multi-class affective state classification highlights the potential of Transformer-based approaches as a promising tool for affective computing in real-world scenarios.

The primary contributions of this study are as follows:

- Implementation of an Innovative Architecture for Affective State Recognition: We propose a Transformer-based model specifically designed for multi-modal, long-term physiological data and optimized for affective state recognition.
- Evaluation Using Real-World Data: The proposed model underwent an extensive assessment utilizing the DAPPER dataset, which includes multi-day recordings of physiological signals from a varied cohort of subjects. The evaluation covered both binary and multi-class classification tasks for affective states, demonstrating the model’s robustness and adaptability.
- Potential Applications: Our findings highlight the feasibility of implementing Transformer-based affective computing systems in real-world settings. This work emphasizes the potential of affective state recognition using wearable sensors, enabling practical applications in everyday life.

## 2. Related Works

### 2.1. Affective State Recognition Using Non-Physiological and Physiological Signals

Affective state recognition has traditionally relied on non-physiological signals, including facial expressions [17], vocal intonations [18], and text [19], as well as physiological signals, such as EEG [20,21,22], heart rate [23], and skin conductance [24]. Each signal modality provides certain benefits, while posing unique obstacles, especially in the context of continuous, real-world affective state monitoring.

Non-physiological signals, including facial expressions and voice attributes, are widely used for the detection of affective states. Facial expression analysis employs techniques like CNNs [25] and SVMs [26] to extract affective signals from facial expressions and movement patterns. Vocal-based affective state recognition relies on acoustic features, including pitch and tone [27], and typically utilizes RNNs [10] or Gaussian mixture models [28] to analyze these temporal dynamics. Nonetheless, non-physiological signals encounter considerable constraints for practical applications, owing to their susceptibility to intentional manipulation and environmental interference. For instance, facial expressions and voice intonations can be consciously altered, making data susceptible to intentional disguising or cultural variations. Moreover, environmental variables like illumination and ambient noise can affect the dependability of these modalities, complicating their use in uncontrolled settings. Consequently, although useful in controlled environments, non-physiological signals may lack the stability required for long-term monitoring of affective states.

Physiological signals, on the other hand, offer a promising alternative for continuous affective state recognition, as they are generally more objective, and provide insights into the arousal and valence dimensions of emotions, which are often difficult to capture using non-physiological data. Wearable devices enable long-term affective state monitoring by recording physiological data such as heart rate, skin conductance, and body movement [2]. With appropriate preprocessing and modeling techniques, these signals can facilitate more nuanced recognition of affective states in real-world settings.

In summary, while both non-physiological and physiological signals can contribute to affective state recognition, physiological signals uniquely enable continuous and unobtrusive monitoring in real-world scenarios. This positions physiological-based approaches as a highly promising direction for future research, with the potential to transform affective computing applications in practical settings.

### 2.2. Wearable Measurement for Affective State Recognition

The increasing prevalence of wearable devices, such as the Apple Watch and Fitbit, has created new opportunities for affective state recognition through continuous physiological data access. These devices record real-time measurements of physiological signals, such as heart rate, skin conductance, and in some cases, EEG, which have shown robust associations with affective states [29]. Wearable-based affective state recognition has the distinct advantage of being non-intrusive and facilitating prolonged monitoring, rendering it particularly appropriate for daily life applications. In contrast to a single-modal signal, the integration of multi-modal signals can enhance the accuracy of affective state recognition by integrating the unique strengths of each modality [30,31].

An increasing number of studies have investigated the integration of various physiological modalities to improve classification accuracy, leveraging the complementary strengths of different signal types to enhance robustness in affective state recognition [32]. Several wearable datasets have contributed to the advancement of multi-modal affective state recognition research, such as the WESAD [33], DAPPER [16], AMIGOS [34] datasets. However, most datasets (e.g., WESAD, AMIGOS, etc.) were collected in laboratory settings, which limits their ability to depict the affective states experienced in real-life situations. Specifically, laboratory environments often lack the complexity and variability of everyday life, which can affect the generalizability of findings to actual daily contexts.

The DAPPER dataset could serve as a benchmark for multi-modal affective state recognition by offering extensive real-world data collected over multiple days. Two recent studies have explored the DAPPER dataset for affective state recognition. Ahmed et al. [35] accomplished depression severity classification and valence–arousal detection for each depression category using diverse machine learning (including SVM, RF, CNN, etc.) approaches based on the DAPPER dataset and achieved an accuracy of 62.9% and 63.9% in high- and low-valence/arousal states for the moderately depressed population, and 61.2% and 56.9% for the severely depressed population. Ahmed et al. [36] further improved binary classification accuracies to 61.55% and 82.75% for arousal and valence scores for a general population using CNN models.

### 2.3. Prior Work on Multi-Modal Affective State Recognition

Traditional machine learning techniques were initially utilized to analyze physiological signals, frequently employing basic models like support vector machines (SVM) and k-nearest neighbors (KNN). For instance, researchers used KNN to classify five different affective states based on the WESAD dataset [37] and binary affective states based on the Amigos dataset [38].

The emergence of deep learning methods has provided more powerful architectures capable of representing multi-modal patterns in physiological data [39]. For example, Dessai et al. [40] employed five pre-trained CNN models for affective state recognition using ECG and GSR signals. Similarly, Tzirakis et al. [41] proposed a multi-modal framework combining a CNN for text modal structures, HRNet for visual modalities, and LSTM networks to capture temporal dynamics in physiological signals. Chen et al. [42] used a hybrid network integrating CNN, LSTM, and graph convolutional network layers for classification tasks. These studies collectively demonstrated the effectiveness of deep learning approaches, with reported accuracies ranging from 69% to well above 95% across different datasets and tasks.

In recent years, the Transformer model [15], which originated in the field of natural language processing (NLP) and was then extended to various fields like image recognition [43] and image segmentation [44], has fundamentally reshaped data modeling and analysis across disciplines. Although contemporary multi-modal models show promising results, they often fail to fully leverage the complex fusion strategies needed to establish cross-modal dependencies. Transformer improves this deficiency by capturing relationships across modalities through a self-attention mechanism, thus improving the model’s robustness and precision. For instance, Ali et al. [45] proposed a Transformer-based method (UBVMT) to process multi-modal data and achieved a binary arousal classification accuracy of 82.9% on a multi-channel EEG dataset. Huang et al. [46] utilized the Transformer model to fuse audio and visual modalities, reporting a classification accuracy of 59.3% for the valence dimension. Cheng et al. [47] applied a hybrid architecture combining a convolutional encoder and a Transformer encoder to classify multi-channel EEG signals, achieving an accuracy of 96.3%. Given the structural similarities between multi-channel EEG and other multi-modal physiological signals, Transformer-based models are expected to exhibit good performance in capturing complex patterns and long-term dependencies.

## 3. Materials and Methods

### 3.1. Dataset Description

We used the DAPPER dataset [16], which recorded the daily dynamic psychological and physiological records of 88 subjects for five consecutive days.

We used experience sampling method (ESM) data for further experiments. Each ESM questionnaire consisted of 20 items, including basic information about daily events, a five-item TIPI-C inventory for self-assessment of personality state, followed by a ten-item positive and negative affect Schedule (PANAS) [48], as well as affective valence and arousal ratings. The ten items selected were upset, hostile, alert, ashamed, inspired, nervous, determined, attentive, afraid, and active. Each item was associated with a 5-point scale.

We also used physiological recordings over five days for analysis, which included the following signals:

- Photoplethysmography (PPG) data. The PPG technique employs green light at a wavelength of 532 nm, with the reflected light intensity measured at a sampling rate of 20 Hz.
- Galvanic skin response (GSR) signals. GSR was measured at the wrist by surface electrodes with conductive gels at a sampling rate of 40 Hz and with a resolution of 0.01 μS.
- Three-axis acceleration data. Three-axis acceleration data were recorded at a sampling rate of 20 Hz.

#### Data Statistics

In the 5-class classification experiment, arousal and valence scores ranging from 1 to 5 corresponded to distinct categories. The distribution of valence and arousal categories is shown in Table 1. We divided the dataset into five classes, ranging from Class 1 (ESM score = 1) to Class 5 (ESM score = 5). The “ESM_Valence” and “ESM_Arousal” rows show the number and proportion of ESM responses falling within each class.

In the binary classification task for the PANAS category, the scores of positive affective items (including inspired, active, determined, and attentive) were added as the total positive score, whereas the scores of negative affective items (including upset, hostile, alert, ashamed, nervous, and afraid) were summed as the total negative score [48]. The category with the higher absolute value between the total positive score and the total negative score was the PANAS category of the instance. Table 2 shows the distributions of the PANAS positive category (Class 1) and the negative (Class 0) category.

### 3.2. Data Preprocessing

**Figure 1:**
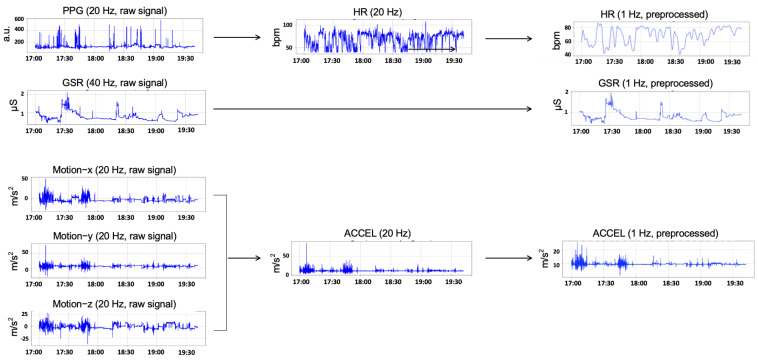
Flow chart of data preprocessing for HR, GSR, and ACCEL signals (data from one representative segment of subject #1004).

We performed the following calculation and preprocessing operations on the multi-modal signals. Figure 1 shows a flow chart of the raw signal and the preprocessed signal for the HR, GSR, and ACCEL signals.

The magnitude of acceleration (ACCEL) was calculated as the square root of the sum of squares of the acceleration in the three orthogonal directions, reflecting the overall motion intensity, with a precision of 1/2048 g (unit of gravity acceleration). The HR signal was derived from the PPG raw data using a joint sparse spectrum reconstruction algorithm [49], implemented in the HuiXin software package (version 201708). The resulting HR data were organized at a 1 Hz sampling rate [50,51]. To ensure relative uniformity across the different signal modalities, the GSR and ACCEL signals were downsampled to match the 1 Hz sampling rate of the HR signal. Specifically, a simple downsampling method was applied, where every 40th sample (for GSR signals) and every 20th sample (for ACCEL signals) was retained from the original signals [52].

For noise reduction, we implemented an adaptive noise cancellation method based on the least mean square algorithm, to handle residual noise that could have interfered with the affective state recognition [53]. Specifically, the algorithm iteratively adjusted the filter coefficients to minimize the mean square error, dynamically reducing the noise in the input signal. The filtered signals were then smoothed using a moving median filter with a kernel size of 3 [54]. The preprocessed signals showed a consistent pattern, as suggested by previous studies [55]. As shown in Figure 1, the signals demonstrated reduced abnormal activities for all signal modalities, as well as reduced high-frequency variations for HR and GSR.

The first 30 min of the physiological data prior to each ESM entry were extracted by matching the timestamps of the ESM with those from the physiological recordings. A total of 3789 segments were extracted, each with both five-class labels and binary labels, for arousal and valence.

### 3.3. Transformer-Based Framework for Multi-Modal Wearable Data

This section will introduce our main framework. Our model aims to effectively capture multi-modal physiological signals to accurately classify affective states. This architecture is based on the Transformer model. The following steps illustrate the construction of our model.

#### 3.3.1. Feature Extraction and Embedding

For each physiological signal, we constructed a separate CNN-based feature extraction network. Presuming that the time series of the input HR signal, GSR signal, and ACCEL signal are $X_{HR}\in R^{T\times d_{HR}}$, $X_{GSR}\in R^{T\times d_{GSR}}$, and $X_{ACCEL}\in R^{T\times d_{ACCEL}}$ respectively. Among these, *T* represents the number of time steps; and $d_{HR}$, $d_{GSR}$, and $d_{ACCEL}$ represent the feature dimensions of each data modality. The extracted features can be represented as $E_{HR}=FeatureExtractor_{HR}\left(X_{HR}\right)$, $E_{GSR}=FeatureExtractor_{GSR}\left(X_{GSR}\right)$ and $E_{ACCEL}=FeatureExtractor_{ACCEL}\left(X_{ACCEL}\right)$, $E_{HR}$, and $E_{GSR}$ and $E_{ACCEL}$ represent the feature expressions of signals.

#### 3.3.2. Multi-Modal Embedding and Concatenation

In multi-modal affective state recognition tasks, the fusion between different signals is important. We concatenated the embedded vectors of HR, GSR, and ACCEL data. These features were then input into the Transformer encoder for joint processing of multi-modal features. Firstly, concatenate $E_{HR}$, $E_{HR}$, and $E_{ACCEL}$ along the feature dimension to obtain the fused multi-modal input representation:

(1) $$E_{concat}=\left[E_{HR};E_{GSR};E_{ACCEL}\right]\in R^{T\times 3d_{E}}$$

Positional encoding *P* is added to $E_{concat}$ to introduce temporal order to the embeddings:

(2) $$E_{input}=E_{concat}+P,$$

where the positional encoding *P* is defined as per the sinusoidal function introduced by Vaswani et al. [15]:

(3) $$P_{\left(i,2j\right)}=sin\frac{i}{10000^{2j/d}},P_{\left(i,2j+1\right)}=cos\frac{i}{10000^{2j/d}},$$

where *i* is the time step, *j* is the embedding dimension, and *d* is the dimensionality of the embeddings.

#### 3.3.3. Transformer Encoder for Multi-Modal Fusion

**Figure 2:**
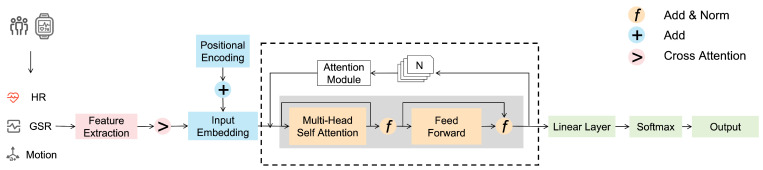
The framework of the proposed architecture.

The Transformer is a model architecture that exclusively utilizes an attention mechanism to establish the global interdependence between input and output. Like most sequence-to-sequence models, Transformer is also an encoder–decoder architecture. However, as physiological recording signals do not have a standard translation, we only use the encoder part. Figure 2 shows the detailed technological process of our Transformer model. The fused input embeddings are passed through a series of Transformer encoder layers, where each layer includes multi-head self-attention and feed-forward layers. The purpose of this module is to learn complex temporal and cross-modal dependencies that contribute to affective state classification. The output from the multi-head attention module undergoes processing by a feed-forward network.

(4) $$H^{l+1}=FeedForward(MultiHead{\left(H\right)}^{\left(l\right)})+H^{\left(l\right)}$$

Among these, $H^{l}$ represents the input of *l* layer. Each attention head calculates attention scores to capture relevant temporal patterns within and across modalities. For each query *Q*, key *K*, and value *V*, the attention mechanism is defined as

(5) $$Attention\left(Q,K,V\right)=Softmax\left(\frac{QK^{T}}{\sqrt{d_{k}}}\right)V,$$

where $d_{k}$ denotes the dimensionality of the keys. Multi-head attention allows the model to attend to different aspects of the signal simultaneously, enhancing the ability to capture diverse patterns. The output from each attention head is concatenated and passed through a linear transformation, represented as

(6) $$MultiHead\left(Q,K,V\right)=Concat\left(head_{1},\ldots ,head_{h}\right)W^{O},$$

where $W^{O}$ is the weight matrix of the output projections.

#### 3.3.4. Classification Layer

The encoded output from the final Transformer layer is input into a classification head, which associates the representations with the affective state labels. This procedure entails a linear layer succeeded by a softmax function to forecast class probabilities:

(7) $$\hat{y}=Softmax\left(W_{out}E_{final}+b_{out}\right),$$

where $W_{out}$ and $b_{out}$ are the weights and bias of the output layer. The predicted label $\hat{y}$ is then compared to the true label *y* using a categorical cross-entropy loss function:

(8) $$L=-\sum_{c=1}^{C}y_{c}log\left(\hat{y_{c}}\right),$$

where *C* is the number of the affective state classes (binary or multi-class).

#### 3.3.5. Evaluation Metrics

We used common classification metrics, including

Accuracy: The proportion of correct predictions across all classes.

Precision: The proportion of true positives among the samples predicted as positive.

Macro Averaged F1 Score: The harmonic mean of precision and recall, providing a balanced measure of accuracy and robustness.

### 3.4. Experiment Settings

We conducted all experiments on eight NVIDIA 1080 GPUs (NVIDIA, Santa Clara, CA, USA), which allowed us to process data efficiently and train the model within a reasonable timeframe. The model was optimized using the Adam optimizer with parameters $\beta_{1}=0.9$, $\beta_{2}=0.999$, and $\epsilon =10^{-8}$. This optimizer was chosen due to its adaptability in handling sparse gradients and its effectiveness in convergence. The learning rate was initialized at 1 $\times \;10^{-3}$ and followed a linear decay schedule to ensure gradual and stable convergence as the training progressed. We set the batch size to 64, which balanced the computational efficiency and stability of the gradient estimates, making it suitable for our dataset. Our model was trained for a total of 100 epochs, with an early stopping criterion applied if the validation performance did not improve over 10 consecutive epochs. This approach mitigated overfitting. To further address overfitting, we applied a dropout rate of 0.2 in the network and introduced L2 regularization with a coefficient of 1 $\times \;10^{-5}$ in the optimizer.

We employed a CNN for feature extraction, utilizing a hidden size of 128, generating a 512-dimensional feature vector as input for the Transformer model. In our experiments, we divided the entire dataset into training and testing sets, with an 8:2 ratio. To avoid possible cross-influence among the different time periods within the same subjects, all data in the training and testing sets were separated by subjects. Our study focused on two main tasks: binary classification based on PANAS scores, and five-class classification based on valence and arousal scores.

In addition, we choose random forest [56], SVM [57] (RBF as kernel function, C = 1.0, gamma = 0.1), AlexNet [58] (5 Convolutional layers and ReLU function), ResNet34 [59], and RNN [60] (128 hidden units) as comparison models.

## 4. Results

The results presented in Table 3 illustrate the binary classification performance based on PANAS score across the different data modalities: HR, GSR, ACCEL, and all three modalities. The accuracy, F1 score, and precision results indicate that the proposed model surpassed the other classifiers within each modality. Notably, the proposed model achieved the highest accuracy and F1 score, reaching an accuracy of 71.50% and an F1 score of 70.38% when using multi-modal data. When using a single data modality, the accuracy of the HR modality was better than the GSR and ACCEL modality data.

**Figure 3:**
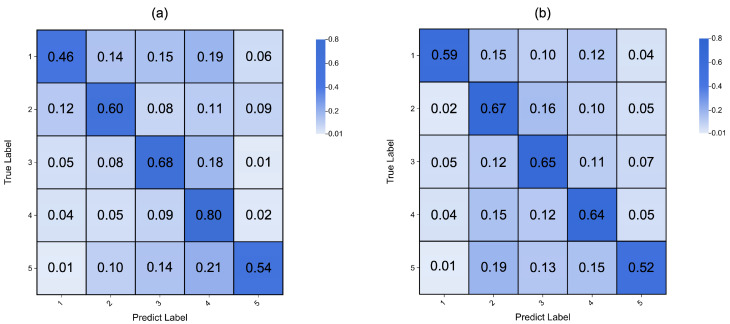
Confusion matrix for 5-class classification tasks. The horizontal axis represents predicted labels, the vertical axis represents true labels, and the number in each cell represents the proportion of each true label being classified into different categories. Figure (**a**) shows a confusion matrix of the valence classification result, and (**b**) shows a confusion matrix of the arousal classification result.

The confusion matrices presented in Figure 3 illustrate the classification performance for valence and arousal across the five classes. The horizontal axis represents the predicted labels, the vertical axis represents the true labels, and the number in each cell represents the proportion of each true label being classified into the different categories. In both matrices, diagonal numbers indicate that the model accurately predicted the true label. For the valence classification, which is shown in Figure 3a, and the arousal dimension, which is shown in Figure 3b, a similar trend is observed, but there was still confusion between some adjacent categories. This suggests that the model could capture the general feature of affective states.

The performance results of the 5-class classification based on valence scores are shown in Table 4. The proposed model with multi-modal data performed the best across all metrics, reaching an accuracy of 60.29% and F1 score of 59.24%, and demonstrating the potential of multi-modal signal fusion. Compared to single-modal data, the RF, SVM, AlexNet, ResNet, and RNN models all showed improvements using multi-modal data.

Table 5 presents the performance of the 5-class classification based on arousal scores. For HR data, our proposed model achieved an accuracy of 50.02%, with an F1 score of 49.31%. For GSR data, it reached an accuracy of 49.35%, with an F1 score of 48.42%. For ACCEL data, the accuracy was 43.52%, with an F1 score of 42.90%. In comparison, the best-performing traditional models, such as RNN, achieved accuracies between 41.18% and 46.78% using single-modal data. When utilizing multi-modal data, the proposed model achieved an accuracy of 61.55% and an F1 score of 60.89%. This highlights the potential of multi-modal data fusion in enhancing affective state recognition.

The results shown in Table 6 display the model results across the various hyperparameter setups. As batch size and inner dimension increase, performance is often enhanced for both arousal and valence classification tasks. The optimal accuracy and F1 scores for valence classification were attained with a batch size of 32 and an inner dimension of 8. The PANAS classification task achieved the best performance when the batch size and inner dimension were equal to 16.

Table 7 compares the performance of the various modality combinations for the arousal, valence, and PANAS classification tasks. The findings illustrate the benefit of employing various modalities for the affective state recognition tasks and that the single modality exhibited a lower performance. Specifically, for the arousal and valence score classification task, the model achieved accuracies of 61.55% and 60.29% separately, which increased by 20.84% and 16.77% compared to using only the ACCEL modality. Pairwise combinations attained better performance, particularly the combination of HR and GSR, achieving an 56.64% accuracy for arousal and 58.93% accuracy for valence. All three tasks achieved the best results when using multi-modal data.

## 5. Discussion and Conclusions

This study shows the feasibility of applying Transformer-based models on multi-modal physiological data (DAPPER) for affective state recognition in everyday situations. The proposed model achieved a binary PANAS classification accuracy of 71.5% and five-class classification accuracies of 60.29% and 61.55% for valence and arousal scores, respectively. The experiments underscored the importance of hyperparameter optimization, including the batch size and inner dimensions. The choice of batch size and inner dimensions influences model training stability and performance. Larger batch sizes may facilitate smoother gradient updates, while the inner dimension settings directly impact the model’s capacity to learn cross-modal relationships. Furthermore, the incorporation of a multi-modal approach surpassed the single-modal performance. This work demonstrates the effectiveness of the Transformer model for practical affective state recognition tasks and highlights the advantages of multi-modal data fusion in improving the performance of wearable affective state recognition systems.

We obtained promising results in the PANAS score classification task. Our model achieved a 71.5% accuracy in binary PANAS categorization, confirming its ability to handle noisy, real-world inputs. Prior works have often been carried out under strictly controlled laboratory conditions. For instance, Nur et al. [61] attained accuracies of 76.33% for differentiating happy, neutral, and sad using PANAS scores in a controlled experimental setting. Chen et al. [62] reported binary classification accuracies varying from 30% to 87.36%, contingent upon the number of features (ranging from 1 to 39) collected during experiments. These works were performed in laboratory settings with minimal noise and multiple sensors, whereas DAPPER was continuously collected in real-world environments, providing a more authentic representation of daily affective states through three data modalities. Although the accuracy scores in our study may not have surpassed those from more controlled experiments, our research demonstrates the effectiveness of Transformer-based patterns in intricate real-world contexts.

The classification results for arousal and valence further demonstrate the potential for reliable affective state recognition in everyday contexts. To allow a more direct comparison with previous binary classification results, we further reorganized our results into a binary version by treating classes 1–3 as one category and 4–5 as the other category for both valence and arousal ratings. The re-organized results yielded an accuracy of 78.6% for valence and 75.85% for arousal, which was overall better and more balanced than the previous results (62.9% and 63.9% for valence and arousal in [35] and 82.75% and 61.55% in [36]). Notably, our five-class classification performance represents an advancement, as this task had not been previously explored with the same approach. Our five-class accuracy of 61.55% and 60.29% based on arousal and valence scored demonstrates a clear improvement, particularly in capturing fine-grained affective states, the strength of our Transformer-based method in handling temporal dependencies and cross-modal data interactions. This choice of five-class classification allowed for better differentiation of subjects’ affective states and represents an important step toward more precise affective state recognition, essential for real-world applications. The findings underscore the efficiency of Transformer-based models as a powerful and novel method for recognizing affective states in everyday situations, especially for managing intricate multi-class tasks that require nuanced affective differentiations.

The experiments with multi-modal data also showed that multi-modal signals, such as HR, GSR, and ACCEL data, made the model work much better than with a single-modal input. Previous studies have shown that single-modal methods do not always capture important affective cues. As an example, Mocanu et al. [63] showed that the accuracy of identifying an affective state rose from 76.42% for a single modality to 87.85% for multi modalities. Although the tasks are different, using multi-modal data can improve classification performance. This is especially true in real life, where feelings are shown through a variety of physiological channels [14]. Our proposed model effectively captures richer affective information by combining multi-modal data, demonstrating the reliability and utility of such an approach for a wide range of affective state recognition tasks.

Despite these promising results, the dataset size and model structure remain limiting factors for large Transformer models. Expanding sample sizes and subject diversity will be crucial for building more promising and generalizable models [64]. The fusion strategy used in this study, based on concatenation, provides a promising baseline. However, more complex fusion strategies [65], such as feature-level fusion or decision-level fusion, could be further explored. In addition, future work could explore more complex feature extraction methods and attention mechanisms, such as cross-attention [66], enabling the model to dynamically prioritize the most relevant modalities and time frames, thereby enhancing its sensitivity to subtle differences between adjacent affective categories. Emerging techniques, such as time-series Transformers and graph convolutional networks [67], could be explored to capture the complex interactions among multi-modal features. Additionally, refining Transformer architectures, particularly with large-scale pre-trained models optimized for multi-modal data [68], could improve the granularity and accuracy of affective state recognition. Furthermore, the integration of emerging sensor technologies, such as wearable EEG or advanced skin sensors, could further expand the diversity of affective signal types.

This method holds great potential for future integration into mental health monitoring and the provision of personalized recommendations. The reliable recognition of affective states in everyday contexts, based on wearable measurements, enables convenient and continuous tracking of affective states in daily life. This approach provides richer and more nuanced individualized data for the clinical diagnosis of mental health issues such as depression and anxiety [69,70]. Wearable devices also facilitate the support of individuals in conducting affective regulation and other types of mental health intervention training in more accessible settings, such as at home [71,72]. Furthermore, the continuous affective recognition of individuals in specific scenarios, such as watching movies or visiting museums, could introduce a new paradigm for user experience evaluation and personalized recommendations [73,74]. By capturing the affective responses in these contexts, we could better understand user engagement and tailor experiences to meet individual needs, enhancing both quality of life and the effectiveness of mental health support.

## Figures and Tables

**Table 1:** The distribution of valence and arousal scores in the DAPPER dataset.

	Class 1	Class 2	Class 3	Class 4	Class 5
ESM_Valence	83 (2%)	613 (16%)	1110 (29%)	1612 (43%)	371 (10%)
ESM_Arousal	318 (8%)	1236 (33%)	998 (26%)	1030 (27%)	207 (6%)

**Table 2:** The distribution of PANAS score in the DAPPER dataset.

	Class 0	Class 1
ESM_PANAS	2090	1699

**Table 3:** Performance of binary classification based on PANAS scores.

Label	Modality	Model	ACC	F1 Score	Precision
PANAS	HR	RF	56.62	53.34	49.56
		SVM	61.98	60.63	57.12
		AlexNet	62.26	61.85	58.27
		ResNet34	61.59	59.72	55.34
		RNN	64.92	62.59	62.44
		Proposed model	**65.26**	**62.61**	**63.19**
	GSR	RF	58.06	56.88	56.90
		SVM	61.63	60.04	57.65
		AlexNet	62.22	60.06	59.41
		ResNet34	62.49	57.09	55.50
		RNN	63.79	62.24	61.22
		Proposed model	**64.39**	**63.34**	**61.93**
	ACCEL	RF	52.86	51.34	46.71
		SVM	55.38	51.03	53.46
		AlexNet	56.77	54.03	**55.41**
		ResNet34	55.94	52.94	49.34
		RNN	54.61	52.17	47.70
		Proposed model	**59.42**	**58.45**	54.68
	Multi-Modal	RF	60.98	57.81	55.37
		SVM	65.48	66.33	61.45
		AlexNet	67.02	65.47	60.36
		ResNet34	66.52	64.33	60.02
		RNN	68.47	64.27	61.10
		Proposed model	**71.50**	**70.38**	**64.26**

The bold numbers indicate the highest performance values for each metric across different methods.

**Table 4:** Performance of 5-class classification based on valence score.

Label	Modality	Model	ACC	F1 Score	Precision
Valence	HR	RF	39.17	38.07	39.93
		SVM	41.87	40.71	43.74
		AlexNet	44.85	44.90	44.70
		ResNet34	44.23	40.21	39.66
		RNN	45.31	44.60	41.40
		Proposed model	**49.38**	**49.42**	**48.89**
	GSR	RF	40.20	39.05	39.71
		SVM	41.13	40.59	41.14
		AlexNet	43.67	41.19	40.10
		ResNet34	46.53	43.68	41.08
		RNN	47.38	44.28	43.89
		Proposed model	**49.09**	**48.30**	**46.65**
	ACCEL	RF	33.11	31.87	31.35
		SVM	35.22	34.75	35.21
		AlexNet	38.47	37.81	36.65
		ResNet34	55.94	52.94	49.34
		RNN	39.33	38.31	38.67
		Proposed model	**40.71**	**40.14**	**41.94**
	Multi-Modal	RF	51.25	49.12	49.10
		SVM	52.34	51.91	51.23
		AlexNet	55.24	54.55	52.47
		ResNet34	56.02	54.52	51.25
		RNN	57.16	55.14	52.49
		Proposed model	**60.29**	**59.24**	**57.67**

The bold numbers indicate the highest performance values for each metric across different methods.

**Table 5:** Performance of 5-class classification based on arousal score.

Label	Modality	Model	ACC	F1 Score	Precision
Arousal	HR	RF	40.51	39.49	39.53
		SVM	42.15	41.82	42.50
		AlexNet	45.37	44.63	43.92
		ResNet34	45.97	43.54	44.23
		RNN	46.25	45.30	44.21
		Proposed model	**50.02**	**49.31**	**48.78**
	GSR	RF	41.73	39.65	40.19
		SVM	41.04	40.45	39.98
		AlexNet	44.12	43.20	42.59
		ResNet34	45.26	43.45	44.16
		RNN	46.78	45.89	45.01
		Proposed model	**49.35**	**48.42**	**47.83**
	ACCEL	RF	32.08	31.22	30.19
		SVM	35.67	34.82	34.39
		AlexNet	39.21	38.47	37.95
		ResNet34	40.20	39.33	38.08
		RNN	41.18	40.27	39.73
		Proposed model	**43.52**	**42.90**	**41.65**
	Multi-Modal	RF	33.11	31.87	31.35
		SVM	52.16	51.87	50.64
		AlexNet	55.48	53.90	52.38
		ResNet34	56.66	55.30	53.84
		RNN	57.32	56.12	54.97
		Proposed model	**61.55**	**60.89**	**57.44**

The bold numbers indicate the highest performance values for each metric across different methods.

**Table 6:** The results of different hyperparameters for the classification task.

Batch Size	Inner Dimension	Arousal_ACC	Arousal_F1	Valence_ACC	Valence_F1	PANAS_ACC	PANAS_F1
8	4	56.43	50.12	57.43	53.03	67.93	66.19
8	8	59.24	57.58	58.46	52.28	68.51	67.12
8	16	58.07	55.80	58.25	52.65	68.97	66.78
16	4	57.23	52.54	58.46	51.82	69.81	67.12
16	8	58.11	53.32	59.57	55.40	70.47	68.18
16	16	58.05	55.26	58.89	54.55	**71.50**	**70.38**
32	4	58.96	53.68	60.03	57.21	71.28	68.40
32	8	**61.55**	**60.89**	60.29	**59.24**	71.32	68.51
32	16	59.80	56.25	**61.06**	56.21	71.22	69.05

The bold numbers indicate the highest performance values for each metric.

**Table 7:** Performance comparison of different modality combinations.

HR	GSR	ACCEL	Arousal_ACC	Arousal_F1	Valence_ACC	Valence_F1	PANAS_ACC	PANAS_F1
✓	×	×	49.38	49.42	50.02	49.31	65.26	62.61
×	✓	×	49.09	48.30	49.35	48.42	64.39	63.34
×	×	✓	40.71	40.14	43.52	42.90	59.42	58.45
✓	✓	×	56.64	53.51	58.93	54.16	68.44	65.38
×	✓	✓	52.57	51.05	53.78	51.37	66.68	64.08
✓	×	✓	53.71	49.39	55.82	52.91	65.95	62.35
✓	✓	✓	**61.55**	**60.89**	**60.29**	**59.24**	**71.50**	**70.38**

The bold numbers indicate the highest performance values for each metric.

## Data Availability

The present study used the publicly available DAPPER dataset. The dataset is available at https://doi.org/10.7303/syn22418021.

## References

[1] Jung T.P., Sejnowski T.J. (2019). Utilizing deep learning towards multi-modal bio-sensing and vision-based affective computing. IEEE Trans. Affect. Comput..

[2] Saganowski S., Perz B., Polak A.G., Kazienko P. (2022). Emotion recognition for everyday life using physiological signals from wearables: A systematic literature review. IEEE Trans. Affect. Comput..

[3] Houben M., Van Den Noortgate W., Kuppens P. (2015). The relation between short-term emotion dynamics and psychological well-being: A meta-analysis. Psychol. Bull..

[4] Hsu J.H., Su M.H., Wu C.H., Chen Y.H. (2021). Speech emotion recognition considering nonverbal vocalization in affective conversations. IEEE/ACM Trans. Audio Speech Lang. Process..

[5] Chen L., Su W., Feng Y., Wu M., She J., Hirota K. (2020). Two-layer fuzzy multiple random forest for speech emotion recognition in human-robot interaction. Inf. Sci..

[6] Siargkas C., Papapanagiotou V., Delopoulos A. (2024). Transportation mode recognition based on low-rate acceleration and location signals with an attention-based multiple-instance learning network. IEEE Trans. Intell. Transp. Syst..

[7] Fu K., Du C., Wang S., He H. (2023). Improved Video Emotion Recognition with Alignment of CNN and Human Brain Representations. IEEE Trans. Affect. Comput..

[8] Wang X., Ma Y., Cammon J., Fang F., Gao Y., Zhang Y. (2023). Self-supervised EEG emotion recognition models based on CNN. IEEE Trans. Neural Syst. Rehabil. Eng..

[9] Fan T., Qiu S., Wang Z., Zhao H., Jiang J., Wang Y., Xu J., Sun T., Jiang N. (2023). A new deep convolutional neural network incorporating attentional mechanisms for ECG emotion recognition. Comput. Biol. Med..

[10] Yadav S.P., Zaidi S., Mishra A., Yadav V. (2022). Survey on machine learning in speech emotion recognition and vision systems using a recurrent neural network (RNN). Arch. Comput. Methods Eng..

[11] Garg D., Verma G.K., Singh A.K. (2024). EEG-based emotion recognition using MobileNet Recurrent Neural Network with time-frequency features. Appl. Soft Comput..

[12] Yang K., Wang C., Gu Y., Sarsenbayeva Z., Tag B., Dingler T., Wadley G., Goncalves J. (2021). Behavioral and physiological signals-based deep multimodal approach for mobile emotion recognition. IEEE Trans. Affect. Comput..

[13] Zhang J., Yin Z., Chen P., Nichele S. (2020). Emotion recognition using multi-modal data and machine learning techniques: A tutorial and review. Inf. Fusion.

[14] Chen S., Tang J., Zhu L., Kong W. (2023). A multi-stage dynamical fusion network for multimodal emotion recognition. Cogn. Neurodyn..

[15] Vaswani A., Shazeer N., Parmar N., Uszkoreit J., Jones L., Gomez A.N., Kaiser Ł., Polosukhin I. (2017). Attention is all you need. Advances in Neural Information Processing Systems.

[16] Shui X., Zhang M., Li Z., Hu X., Wang F., Zhang D. (2021). A dataset of daily ambulatory psychological and physiological recording for emotion research. Sci. Data.

[17] Krumhuber E.G., Skora L.I., Hill H.C., Lander K. (2023). The role of facial movements in emotion recognition. Nat. Rev. Psychol..

[18] Chen W., Xing X., Chen P., Xu X. (2024). Vesper: A compact and effective pretrained model for speech emotion recognition. IEEE Trans. Affect. Comput..

[19] Meng T., Shou Y., Ai W., Yin N., Li K. (2024). Deep imbalanced learning for multimodal emotion recognition in conversations. IEEE Trans. Artif. Intell..

[20] Li D., Xie L., Wang Z., Yang H. (2023). Brain emotion perception inspired EEG emotion recognition with deep reinforcement learning. IEEE Trans. Neural Netw. Learn. Syst..

[21] Ju X., Li M., Tian W., Hu D. (2024). EEG-based emotion recognition using a temporal-difference minimizing neural network. Cogn. Neurodyn..

[22] Pamungkas Y., Wibawa A.D., Rais Y. (2022). Classification of emotions (positive-negative) based on eeg statistical features using rnn, lstm, and bi-lstm algorithms. Proceedings of the 2022 2nd International Seminar on Machine Learning, Optimization, and Data Science (ISMODE).

[23] Shu L., Yu Y., Chen W., Hua H., Li Q., Jin J., Xu X. (2020). Wearable emotion recognition using heart rate data from a smart bracelet. Sensors.

[24] Chatterjee D., Gavas R., Saha S.K. (2022). Exploring skin conductance features for cross-subject emotion recognition. Proceedings of the 2022 IEEE Region 10 Symposium (TENSYMP).

[25] Ozdemir M.A., Elagoz B., Alaybeyoglu A., Sadighzadeh R., Akan A. (2019). Real time emotion recognition from facial expressions using CNN architecture. Proceedings of the 2019 Medical Technologies Congress (TIPTEKNO).

[26] Michel P., El Kaliouby R. Real time facial expression recognition in video using support vector machines. Proceedings of the 5th International Conference on Multimodal Interfaces.

[27] Noroozi F., Sapiński T., Kamińska D., Anbarjafari G. (2017). Vocal-based emotion recognition using random forests and decision tree. Int. J. Speech Technol..

[28] Navyasri M., RajeswarRao R., DaveeduRaju A., Ramakrishnamurthy M. (2018). Robust features for emotion recognition from speech by using Gaussian mixture model classification. Proceedings of the Information and Communication Technology for Intelligent Systems (ICTIS 2017)-Volume 2.

[29] Gouizi K., Bereksi Reguig F., Maaoui C. (2011). Emotion recognition from physiological signals. J. Med Eng. Technol..

[30] Ezzameli K., Mahersia H. (2023). Emotion recognition from unimodal to multimodal analysis: A review. Inf. Fusion.

[31] Banik S., Kumar H., Ganapathy N., Swaminathan R. (2024). Exploring Central-Peripheral Nervous System Interaction Through Multimodal Biosignals: A Systematic Review. IEEE Access.

[32] Zhang S., Yang Y., Chen C., Zhang X., Leng Q., Zhao X. (2024). Deep learning-based multimodal emotion recognition from audio, visual, and text modalities: A systematic review of recent advancements and future prospects. Expert Syst. Appl..

[33] Schmidt P., Reiss A., Duerichen R., Marberger C., Van Laerhoven K. Introducing wesad, a multimodal dataset for wearable stress and affect detection. Proceedings of the 20th ACM International Conference on Multimodal Interaction.

[34] Miranda-Correa J.A., Abadi M.K., Sebe N., Patras I. (2018). Amigos: A dataset for affect, personality and mood research on individuals and groups. IEEE Trans. Affect. Comput..

[35] Ahmed A., Ramesh J., Ganguly S., Aburukba R., Sagahyroon A., Aloul F. (2022). Investigating the feasibility of assessing depression severity and valence-arousal with wearable sensors using discrete wavelet transforms and machine learning. Information.

[36] Ahmed A., Ramesh J., Ganguly S., Aburukba R., Sagahyroon A., Aloul F. (2023). Evaluating multimodal wearable sensors for quantifying affective states and depression with neural networks. IEEE Sens. J..

[37] Bajpai D., He L. (2020). Evaluating knn performance on wesad dataset. Proceedings of the 2020 12th International Conference on Computational Intelligence and Communication Networks (CICN).

[38] Sepúlveda A., Castillo F., Palma C., Rodriguez-Fernandez M. (2021). Emotion recognition from ECG signals using wavelet scattering and machine learning. Appl. Sci..

[39] Khaleghi A., Shahi K., Saidi M., Babaee N., Kaveh R., Mohammadian A. (2024). Linear and nonlinear analysis of multimodal physiological data for affective arousal recognition. Cogn. Neurodyn..

[40] Dessai A., Virani H. (2023). Emotion Classification Based on CWT of ECG and GSR Signals Using Various CNN Models. Electronics.

[41] Tzirakis P., Chen J., Zafeiriou S., Schuller B. (2021). End-to-end multimodal affect recognition in real-world environments. Inf. Fusion.

[42] Chen J., Hu Y., Garg L., Gadekallu T.R., Srivastava G., Wang W. (2024). Graph Enhanced Low-Resource ECG Representation Learning for Emotion Recognition Based on Wearable Internet of Things. IEEE Internet Things J..

[43] Dosovitskiy A. (2020). An image is worth 16x16 words: Transformers for image recognition at scale. arXiv.

[44] Valanarasu J.M.J., Oza P., Hacihaliloglu I., Patel V.M. (2021). Medical transformer: Gated axial-attention for medical image segmentation. Proceedings of the Medical Image Computing and Computer Assisted Intervention–MICCAI 2021: 24th International Conference.

[45] Ali K., Hughes C.E. (2023). A Unified Transformer-based Network for Multimodal Emotion Recognition. arXiv.

[46] Huang J., Tao J., Liu B., Lian Z., Niu M. (2020). Multimodal transformer fusion for continuous emotion recognition. Proceedings of the ICASSP 2020—2020 IEEE International Conference on Acoustics, Speech and Signal Processing (ICASSP).

[47] Cheng C., Liu W., Fan Z., Feng L., Jia Z. (2024). A novel transformer autoencoder for multi-modal emotion recognition with incomplete data. Neural Netw..

[48] Watson D., Clark L.A., Tellegen A. (1988). Development and validation of brief measures of positive and negative affect: The PANAS scales. J. Personal. Soc. Psychol..

[49] Zhang Z. (2015). Photoplethysmography-based heart rate monitoring in physical activities via joint sparse spectrum reconstruction. IEEE Trans. Biomed. Eng..

[50] Qu Z., Chen J., Li B., Tan J., Zhang D., Zhang Y. (2020). Measurement of high-school students’ trait math anxiety using neurophysiological recordings during math exam. IEEE Access.

[51] Zhang Y., Qin F., Liu B., Qi X., Zhao Y., Zhang D. (2018). Wearable neurophysiological recordings in middle-school classroom correlate with students’ academic performance. Front. Hum. Neurosci..

[52] Pasquini L., Noohi F., Veziris C.R., Kosik E.L., Holley S.R., Lee A., Brown J.A., Roy A.R., Chow T.E., Allen I. (2023). Dynamic autonomic nervous system states arise during emotions and manifest in basal physiology. Psychophysiology.

[53] Ghosh A., Torres J.M.M., Danieli M., Riccardi G. (2015). Detection of essential hypertension with physiological signals from wearable devices. Proceedings of the 2015 37th Annual International Conference of the IEEE Engineering in Medicine and Biology Society (EMBC).

[54] Bakker J., Pechenizkiy M., Sidorova N. (2011). What’s your current stress level? Detection of stress patterns from GSR sensor data. Proceedings of the 2011 IEEE 11th International Conference on Data Mining Workshops.

[55] Iadarola G., Poli A., Spinsante S. (2021). Analysis of galvanic skin response to acoustic stimuli by wearable devices. Proceedings of the 2021 IEEE International Symposium on Medical Measurements and Applications (MeMeA).

[56] Breiman L. (2001). Random forests. Mach. Learn..

[57] Chen P.H., Lin C.J., Schölkopf B. (2005). A tutorial on *ν*-support vector machines. Appl. Stoch. Model. Bus. Ind..

[58] Krizhevsky A., Sutskever I., Hinton G.E. (2017). ImageNet classification with deep convolutional neural networks. Commun. ACM.

[59] He K., Zhang X., Ren S., Sun J. Deep residual learning for image recognition. Proceedings of the IEEE Conference on Computer Vision and Pattern Recognition.

[60] McLaughlin N., Del Rincon J.M., Miller P. Recurrent convolutional network for video-based person re-identification. Proceedings of the IEEE Conference on Computer Vision and Pattern Recognition.

[61] Nur Z.K., Wijaya R., Wulandari G.S. (2024). Optimizing Emotion Recognition with Wearable Sensor Data: Unveiling Patterns in Body Movements and Heart Rate through Random Forest Hyperparameter Tuning. arXiv.

[62] Chen T.H., Chen S.J., Lee S.E., Lee Y.J. (2023). Classification of high mental workload and emotional statuses via machine learning feature extractions in gait. Int. J. Ind. Ergon..

[63] Mocanu B., Tapu R., Zaharia T. (2023). Multimodal emotion recognition using cross modal audio-video fusion with attention and deep metric learning. Image Vis. Comput..

[64] Yu Y., Zhuang Y., Zhang J., Meng Y., Ratner A.J., Krishna R., Shen J., Zhang C. (2024). Large language model as attributed training data generator: A tale of diversity and bias. Advances in Neural Information Processing Systems.

[65] Praveen R.G., Cardinal P., Granger E. (2023). Audio–visual fusion for emotion recognition in the valence–arousal space using joint cross-attention. IEEE Trans. Biom. Behav. Identity Sci..

[66] Jia L., Ma T., Rong H., Al-Nabhan N. (2023). Affective region recognition and fusion network for target-level multimodal sentiment classification. IEEE Trans. Emerg. Top. Comput..

[67] Yun S., Jeong M., Kim R., Kang J., Kim H.J. (2019). Graph transformer networks. Advances in Neural Information Processing Systems.

[68] Chen H., Wang Y., Guo T., Xu C., Deng Y., Liu Z., Ma S., Xu C., Xu C., Gao W. Pre-trained image processing transformer. Proceedings of the IEEE/CVF Conference on Computer Vision and Pattern Recognition.

[69] Fedor S., Lewis R., Pedrelli P., Mischoulon D., Curtiss J., Picard R.W. (2023). Wearable technology in clinical practice for depressive disorder. N. Engl. J. Med..

[70] Shui X., Xu H., Tan S., Zhang D. (2025). Depression recognition using daily wearable-derived physiological data. Sensors.

[71] Fodor K., Balogh Z., Molnár G. (2023). Real-time emotion recognition in smart homes. Proceedings of the 2023 IEEE 17th International Symposium on Applied Computational Intelligence and Informatics (SACI).

[72] Liu S., Gao P., Li Y., Fu W., Ding W. (2023). Multi-modal fusion network with complementarity and importance for emotion recognition. Inf. Sci..

[73] Duan S., Wang Z., Wang S., Chen M., Zhang R. (2024). Emotion-aware interaction design in intelligent user interface using multi-modal deep learning. arXiv.

[74] Cosoli G., Poli A., Scalise L., Spinsante S. (2021). Heart rate variability analysis with wearable devices: Influence of artifact correction method on classification accuracy for emotion recognition. Proceedings of the 2021 IEEE International Instrumentation and Measurement Technology Conference (I2MTC).

